# Strain-Level Differences in Porphyrin Production and Regulation in *Propionibacterium acnes* Elucidate Disease Associations

**DOI:** 10.1128/mSphere.00023-15

**Published:** 2016-02-10

**Authors:** Tremylla Johnson, Dezhi Kang, Emma Barnard, Huiying Li

**Affiliations:** a Department of Molecular and Medical Pharmacology, Crump Institute for Molecular Imaging, David Geffen School of Medicine, UCLA, Los Angeles, California, USA; b UCLA-DOE Institute for Genomics and Proteomics, Los Angeles, California, USA; University of Kentucky

**Keywords:** porphyrin, vitamin B_12_, *Propionibacterium acnes*, strain, acne, 5-aminolevulinic acid, levulinic acid

## Abstract

*Propionibacterium acnes* is an important skin commensal, but it is also considered a pathogenic factor in several diseases including acne vulgaris, the most common skin disease. While previous studies have revealed *P. acnes* strain-level differences in health and disease associations, the underlying molecular mechanisms remain unknown. Recently, we demonstrated that vitamin B_12_ supplementation increases *P. acnes* production of porphyrins, a group of proinflammatory metabolites important in acne development (D. Kang, B. Shi, M. C. Erfe, N. Craft, and H. Li, Sci. Transl. Med. 7:293ra103, 2015, doi:10.1126/scitranslmed.aab2009). In this study, we compared the porphyrin production and regulation of multiple *P. acnes* strains. We revealed that acne-associated type IA-2 strains inherently produced significantly higher levels of porphyrins, which were further enhanced by vitamin B_12_ supplementation. On the other hand, health-associated type II strains produced low levels of porphyrins and did not respond to vitamin B_12_. Using a small-molecule substrate and inhibitor, we demonstrated that porphyrin biosynthesis was modulated at the metabolic level. We identified a repressor gene (*deoR*) of porphyrin biosynthesis that was carried in all health-associated type II strains, but not in acne-associated type IA-2 strains. The expression of *deoR* suggests additional regulation of porphyrin production at the transcriptional level in health-associated strains. Our findings provide one potential molecular mechanism for the different contributions of *P. acnes* strains to skin health and disease and support the role of vitamin B_12_ in acne pathogenesis. Our study emphasizes the importance of understanding the role of the commensal microbial community in health and disease at the strain level and suggests potential utility of health-associated *P. acnes* strains in acne treatment.

**IMPORTANCE** *Propionibacterium acnes* is a dominant bacterium residing on skin, and it has been thought to play a causal role in several diseases including acne, a common skin disease affecting more than 80% of people worldwide. While specific strains of *P. acnes* have been associated with either disease or healthy skin, the mechanisms remain unclear. Recently, we showed that vitamin B_12_ supplementation increased porphyrin production in *P. acnes*, leading to acne development (D. Kang, B. Shi, M. C. Erfe, N. Craft, and H. Li, Sci. Transl. Med. 7:293ra103, 2015, doi:10.1126/scitranslmed.aab2009). Here, we reveal that the levels of porphyrin production and vitamin B_12_ regulation are different between acne- and health-associated strains, suggesting a potential molecular mechanism for disease-associated strains in acne pathogenesis and for health-associated strains in skin health. This study highlights the importance of understanding the strain-level differences of the human microbiota in disease pathogenesis. Our findings also suggest the porphyrin biosynthesis pathway as a candidate drug target and use of health-associated strains as potential probiotics in novel acne therapeutics.

## Abstract (precis)

*Propionibacterium acnes* is a dominant bacterium residing on skin, and it has been thought to play a causal role in several diseases including acne, a common skin disease affecting more than 80% of people worldwide. While specific strains of *P. acnes* have been associated with either disease or healthy skin, the mechanisms remain unclear. Recently, we showed that vitamin B_12_ supplementation increased porphyrin production in *P. acnes*, leading to acne development (D. Kang, B. Shi, M. C. Erfe, N. Craft, and H. Li, Sci. Transl. Med. 7:293ra103, 2015, doi:10.1126/scitranslmed.aab2009). Here, we reveal that the levels of porphyrin production and vitamin B_12_ regulation are different between acne- and health-associated strains, suggesting a potential molecular mechanism for disease-associated strains in acne pathogenesis and for health-associated strains in skin health. This study highlights the importance of understanding the strain-level differences of the human microbiota in disease pathogenesis. Our findings also suggest the porphyrin biosynthesis pathway as a candidate drug target and use of health-associated strains as potential probiotics in novel acne therapeutics.

## INTRODUCTION

*Propionibacterium acnes* is a major commensal bacterium residing on human skin. It plays important roles in maintaining skin health, but it has also been implicated in the pathogenesis of several diseases and infections, including sarcoidosis, SAPHO (synovitis, acne, pustulosis, hyperostosis, and osteitis) syndrome, endodontic lesions, eye infections, prosthetic joint infections, prostate cancer, and acne vulgaris (commonly called acne) (1–4). Acne is the most common skin disease, affecting more than 80% of adolescents and young adults worldwide (5, 6). Despite the clinical importance of the disease (7, 8), the etiology of acne is not yet clear. *P. acnes* dominates the skin of both acne patients and healthy individuals, and thus, its role in acne pathogenesis has not been well understood. Previously, culture-based and 16S rRNA metagenomic studies identified *P. acnes* strains that were associated with either acne or healthy skin (9–12). Genome comparison of a large number of strains revealed key genetic differences among *P. acnes* lineages (11, 13, 14). Type IA-2 (primarily ribotype 4 [RT4] and RT5) strains have been associated with acne. They harbor extra genomic elements encoding multiple virulence genes. On the other hand, type II strains, in particular RT6 and some RT2 strains, have rarely been found in acneic skin and thus are defined as health-associated strains in the context of acne (10–12, 15). Type II strains carry clustered regularly interspaced short palindromic repeat (CRISPR) elements, which may prevent these strains from acquiring virulence genes from phage or other foreign DNA. The presence of these genetic elements partly explains how different *P. acnes* strains may play roles in health or disease; however, the molecular mechanisms underlying *P. acnes* strain-level differences in health and disease associations remain to be elucidated.

To date, limited information exists about *P. acnes* strain-level differences beyond the genomic variations. Molecules secreted by *P. acnes*, such as proteases, lipases, hemolysins, and porphyrins, can degrade host tissue and have been suggested as causal factors in acne (16–20). Porphyrins can generate reactive oxygen species and induce inflammation in keratinocytes (21–23). Previous studies have shown correlations between acne severity and the concentrations of bacterium-derived porphyrins in the hair follicle. Increased levels of porphyrins were observed in acneic skin compared to healthy skin, as well as in acne lesions compared to nonlesional sites of acne patients (24–26). Consistently, a reduction in porphyrin levels was observed in acne patients who positively responded to acne treatment, while those who did not respond to acne treatment exhibited unchanged or increased levels of bacterial porphyrins on their skin (27–29).

Vitamin B_12_ has been suggested to induce acne (18, 30–33). In propionibacteria, the vitamin B_12_ and porphyrin biosynthesis pathways are inversely correlated (18, 34). Our recent study suggested that vitamin B_12_ supplementation repressed its own biosynthesis in *P. acnes*, resulting in increased porphyrin production, and led to acne development in a subset of individuals (18).

In this study, we compared the porphyrin production and regulation between acne-associated type IA-2 strains and health-associated type II strains to investigate a potential molecular link between porphyrin production and disease association of various *P. acnes* strains.

## RESULTS

### Acne-associated *P. acnes* strains produced significantly more porphyrins than health-associated strains.

To investigate whether different *P. acnes* strains produce the same porphyrin species, we first characterized the types of porphyrins secreted by multiple *P. acnes* strains using mass spectrometry. Four acne-associated type IA-2 strains, HL053PA1 (RT4), HL045PA1 (RT4), HL043PA1 (RT5), and HL043PA2 (RT5), and three health-associated type II strains, HL001PA1 (RT2), HL103PA1 (RT2), and HL042PA3 (RT6), were examined (Table 1). We found that there was no difference in the porphyrin species produced by these *P. acnes* strains (see Fig. S1 in the supplemental material). Consistent with previous studies (35–37), coproporphyrin III was the dominant porphyrin isomer produced by all strains ([M+H]^+^ =655.3). Minimal amounts of coproporphyrin I were also detected.

10.1128/mSphere.00023-15.1 Figure S1  Coproporphyrin was the dominant porphyrin isoform produced by *P. acnes*. Porphyrins secreted by *P. acnes* have a mass spectrum characteristic of the monoisotopic coproporphyrin isoform ([M + H]^+^ = 655.3). The doubly charged parent ion was also observed ([M + 2H]^2+^ = 328.2). Download Figure S1, PDF file, 0.1 MB. Copyright © 2016 Johnson et al. 2016 Johnson et al. This content is distributed under the terms of the Creative Commons Attribution 4.0 International license.

**TABLE 1:** *P. acnes* strains used in this study

Association or type	Lineage^a^	Phylogroup^b^	Strain	Ribotype^a^	Clonal complex (sequence type by Belfast MLST_8_)^b^	Clonal complex (sequence type by Aarhus MLST_9_)^c^
Acne-associated	IA-2	IA_1_	HL053PA1	RT4	CC3 (ST3)	CC3 (ST3)
			HL045PA1	RT4	CC3 (ST17)	CC3 (ST3)
			HL043PA1	RT5	CC3 (ST3)	CC3 (ST58)
			HL043PA2	RT5	CC3 (ST3)	CC3 (ST58)
Health-associated	II	II	HL001PA1	RT2	CC72 (ST30)	CC60 (ST60)
			HL103PA1	RT2	CC6 (ST25)	CC60 (ST60)
			HL042PA3	RT6	CC6 (ST7)	CC60 (ST73)
Type I with *deoR*	I	IA_1_	HL025PA1	RT1	CC4 (ST4)	CC28 (ST27)

^a^ As described by Fitz-Gibbon et al. (11).

^b^ As described by McDowell et al. (10).

^c^ As described by Lomholt and Kilian (12).

**FIG 1:**
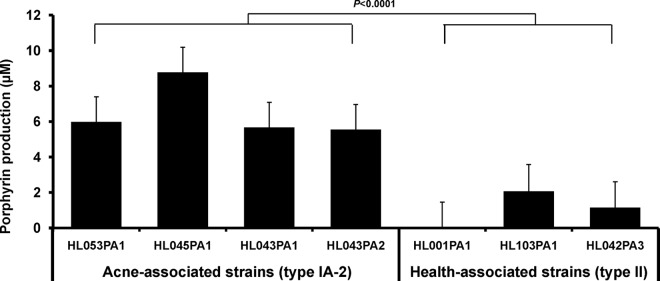
Acne-associated type IA-2 *P. acnes* strains produced significantly more porphyrins than health-associated type II strains. Each bar represents the porphyrins produced by a strain normalized to bacterial culture density. The means plus standard errors (error bars) of data obtained from at least three independent experiments with at least three replicates each are shown.

To determine whether *P. acnes* strains produce different amounts of porphyrins, we next quantified the secreted porphyrins in the seven *P. acnes* strains. The average porphyrin level produced by acne-associated type IA-2 strains was 6.5 µM (5.6 to 8.8 µM), which was significantly greater than that produced by health-associated type II strains, 1.1 µM (0 to 2.1 µM) (*P* < 0.0001) (Fig. 1). Notably, the RT2 strain HL001PA1 produced no detected porphyrins in all experiments. This strain belongs to clonal complex 72 (CC72) based on the Belfast MLST_8_ (multilocus sequence typing using eight housekeeping genes) scheme (Table 1). McDowell et al. reported that strains from CC72 were isolated from healthy skin (10). This is consistent with our finding.

### Vitamin B_12_ supplementation significantly increased porphyrin production in acne-associated strains, but not in health-associated strains.

**FIG 2:**
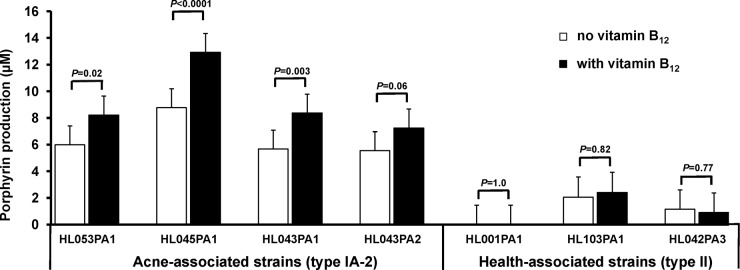
Vitamin B_12_ supplementation significantly increased porphyrin production in acne-associated type IA-2 strains, but not in health-associated type II strains. *P. acnes* strains were cultured in medium with or without the addition of 10 µg/ml vitamin B_12_. Each bar represents the porphyrins produced by a strain normalized to bacterial culture density. The means plus standard errors (error bars) of data obtained from at least three independent experiments with at least three replicates each are shown.

To investigate whether vitamin B_12_ modulates *P. acnes* porphyrin production in a strain-specific manner, we compared the levels of porphyrins produced by different *P. acnes* strains with and without vitamin B_12_ supplementation. We found that vitamin B_12_ supplementation led to increased porphyrin production in all tested acne-associated type IA-2 strains (average porphyrin level increasing from 6.5 µM to 9.2 µM), with statistical significance in three of the four tested strains (*P* ≤ 0.02) (Fig. 2). In contrast, vitamin B_12_ supplementation had no significant effect on porphyrin production in health-associated type II strains (average porphyrin level remaining at 1.1 µM) (*P* ≥ 0.77). The porphyrin levels in strain HL001PA1 remained undetected. To confirm our results, we tested porphyrin production, with and without the addition of vitamin B_12_, of three additional type II strains, HL110PA3 (RT6), HL106PA1 (RT2), and HL050PA2 (RT1). We found that these strains produced similarly low levels of porphyrins (1.4 ± 1.2 µM) and that the production level was not significantly affected by vitamin B_12_ supplementation. Our data suggest that vitamin B_12_ modulates porphyrin production in acne-associated type IA-2 strains, but not in health-associated type II strains, indicating a molecular link between *P. acnes* strain composition of the skin microbiota and the observation that vitamin B_12_ induces acne in a subset of individuals.

### Vitamin B_12_ supplementation repressed vitamin B_12_ biosynthesis gene expression.

**FIG 3:**
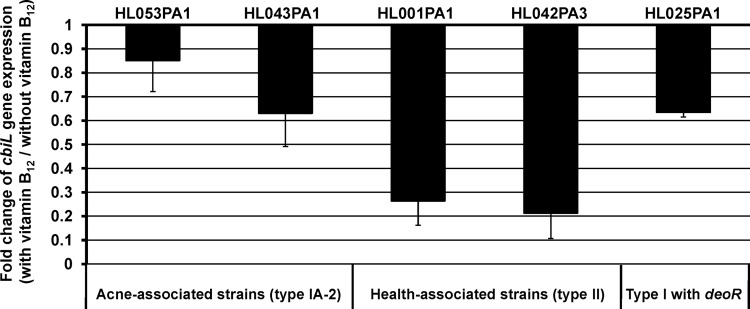
Vitamin B_12_ supplementation repressed the expression of a vitamin B_12_ biosynthesis gene, *cbiL*. The expression level of *cbiL* was quantified by qRT-PCR from *P. acnes* strains cultured with or without the addition of 10 µg/ml vitamin B_12_. Strains of types IA-2 and II and an RT1 strain carrying *deoR*, HL025PA1, are shown. Each bar represents the fold change in gene expression of *cbiL* in cultures with vitamin B_12_ supplementation compared to cultures without supplementation. The means plus standard deviations (error bars) of data obtained from at least two independent experiments with at least three replicates each are shown.

To determine whether vitamin B_12_ affects porphyrin production via repression of its own biosynthesis in *P. acnes*, we performed quantitative reverse transcription-PCR (qRT-PCR) to measure the expression level of *cbiL*, a gene in the vitamin B_12_ biosynthesis pathway. The *cbiL* gene encodes precorrin-2 C-20-methyltransferase, a key enzyme involved in corrin ring formation in the vitamin B_12_ biosynthesis pathway. We found that the addition of vitamin B_12_ to *P. acnes* cultures resulted in the downregulation of *cbiL* gene expression, with average fold changes of 0.74 in acne-associated type IA-2 strains and 0.24 in health-associated type II strains (Fig. 3). The downregulation of *cbiL* gene expression is consistent with the previous finding that vitamin B_12_ supplementation repressed the vitamin B_12_ biosynthesis pathway (18), leading to increased porphyrin production. The expression of *cbiL* was downregulated in both type IA-2 and type II strains, despite our above observation that the porphyrin production in type II strains was unaffected by vitamin B_12_ supplementation. This suggests that additional mechanisms are involved in inhibiting porphyrin production in health-associated type II strains.

### Addition of 5-aminolevulinic acid increased porphyrin production, which was further enhanced by vitamin B_12_.

**FIG 4:**
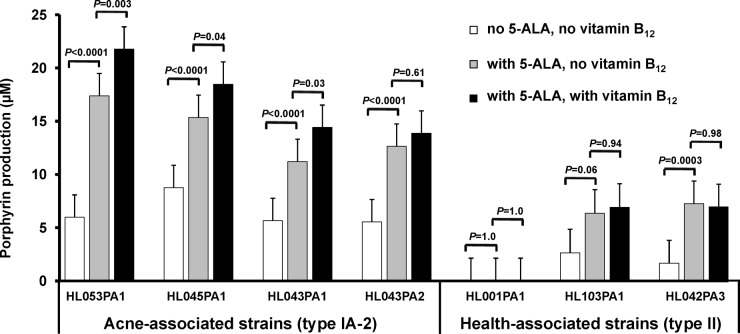
5-ALA increased porphyrin production, which was further enhanced by vitamin B_12_ supplementation in acne-associated type IA-2 strains. *P. acnes* strains were cultured in medium with substrate 5-ALA (0.1 mg/ml) (grey bars) or without 5-ALA (white bars) or with both 5-ALA and vitamin B_12_ (10 µg/ml) added (black bars). 5-ALA significantly increased porphyrin production in acne-associated type IA-2 strains (*P* < 0.0001) and in health-associated type II strains (*P* ≤ 0.06, except for HL001PA1). Vitamin B_12_ supplementation further increased porphyrin production in the presence of 5-ALA in acne-associated type IA-2 strains, but not in health-associated type II strains. Each bar represents the porphyrins produced by each strain normalized to the bacterial culture density. The means plus standard errors (error bars) of data obtained from at least three independent experiments with at least three replicates each are shown.

The porphyrin and vitamin B_12_ biosynthesis pathways in *P. acnes* share the same initial enzymatic steps and a common precursor, 5-aminolevulinic acid (5-ALA), the substrate of the rate-limiting enzyme, porphobilinogen synthase. To investigate whether porphyrin production can be promoted by increasing the availability of 5-ALA, we compared the porphyrin production levels of *P. acnes* strains with and without addition of the substrate. Additionally, we examined whether vitamin B_12_ has an additive effect on porphyrin production when supplemented in combination with 5-ALA. Upon substrate addition only, porphyrin production was significantly increased by average values of 2.2-fold in acne-associated type IA-2 strains (all *P* < 0.0001) and 3.4-fold in health-associated type II strains (*P* ≤ 0.06, except for strain HL001PA1) compared to untreated controls (Fig. 4). Strain HL001PA1 consistently produced no detected porphyrins, even upon addition of 5-ALA. Supplementation of 5-ALA in combination with vitamin B_12_ further enhanced porphyrin production in acne-associated type IA-2 strains (1.2-fold increase compared to 5-ALA only) with statistical significance in three of the four tested strains (*P* ≤ 0.04), but not in health-associated type II strains (*P* ≥ 0.94). This is consistent with our earlier finding that type II strains did not respond to vitamin B_12_ in their porphyrin production (Fig. 2). Our data suggest that the metabolic influx of substrates influences porphyrin production in *P. acnes*. The modulation of porphyrin production by vitamin B_12_ supplementation is specific to acne-associated type IA-2 strains, but not health-associated type II strains.

### Small-molecule inhibitor reduced porphyrin production in *P. acnes*, and its inhibition was counteracted by vitamin B_12_ supplementation.

**FIG 5:**
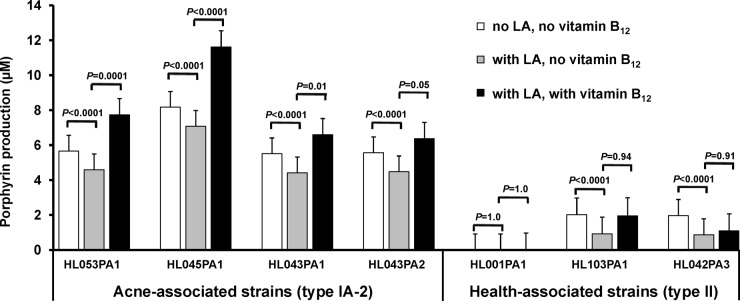
Small-molecule inhibitor reduced porphyrin production in *P. acnes*, and its inhibition was counteracted by vitamin B_12_ supplementation in acne-associated type IA-2 strains. *P. acnes* strains were cultured in medium with inhibitor LA (0.1 mg/ml) (grey bars) or without LA (white bars) or with both LA and vitamin B_12_ (10 µg/ml) added (black bars). LA significantly reduced porphyrin biosynthesis in all strains except for HL001PA1 (*P* < 0.0001). Vitamin B_12_ supplementation counteracted the inhibition of porphyrin biosynthesis by LA in acne-associated type IA-2 strains, but not in health-associated type II strains. Each bar represents the porphyrins produced by each strain normalized to the bacterial culture density. The means plus standard errors (error bars) of data obtained from at least three independent experiments with at least three replicates each are shown.

To further demonstrate that porphyrin production can be modulated at the metabolic level, we investigated the effect of a small-molecule inhibitor, levulinic acid (LA), on porphyrin biosynthesis in *P. acnes* strains. Additionally, we examined whether vitamin B_12_ counteracts the effect of LA on porphyrin production when supplemented in combination with LA. LA is an analog of 5-ALA and has been shown in *Pseudomonas aeruginosa* to inhibit the enzymatic activity of porphobilinogen synthase (38), blocking the metabolic influx to the porphyrin and vitamin B_12_ biosynthesis pathways. We examined a range of concentrations of LA from 0.1 mg/ml to 1.0 mg/ml and found that at the concentration of 0.1 mg/ml, LA did not significantly affect bacterial growth, thus making the measurements of porphyrin production experimentally feasible. LA (0.1 mg/ml) significantly reduced porphyrin production in all strains except for strain HL001PA1 with an average of 30% reduction compared to untreated cultures (*P* < 0.0001) (Fig. 5). However, in acne-associated type IA-2 strains, there was no significant reduction of porphyrins when supplemented in combination with 10 µg/ml vitamin B_12_. Vitamin B_12_ supplementation counteracted inhibition of porphyrin biosynthesis by LA specifically in acne-associated type IA-2 strains, but not in health-associated type II strains. This is consistent with our above findings (Fig. 2 and 4) and further supports our conclusions that vitamin B_12_ modulates porphyrin production in acne-associated type IA-2 strains and that porphyrin production can be modulated at the metabolic level. Our data also suggest that LA is an effective inhibitor of porphyrin biosynthesis in *P. acnes* and that the porphyrin biosynthesis pathway can be a potential therapeutic target for new acne treatments.

### Health-associated strains harbor a porphyrin biosynthesis repressor gene, *deoR*.

**FIG 6:**
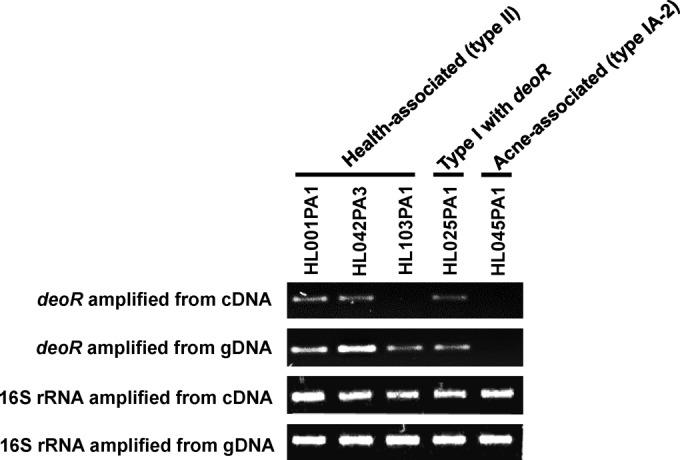
Health-associated strains carried and expressed *deoR*, a repressor gene in the porphyrin biosynthesis operon. *deoR* amplification from the cDNA and genomic DNA (gDNA) samples of multiple strains is shown in the gel image. 16S rRNA gene was used as a positive control. Strain HL045PA1, which is an acne-associated type IA-2 strain and does not carry *deoR*, is shown as a negative control.

To identify potential molecular mechanisms that can explain differences in porphyrin levels produced by acne- and health-associated *P. acnes* strains, we compared the porphyrin biosynthesis gene operon (*hem*) of 82 *P. acnes* strains (13), including the strains tested in this study. We found that all health-associated type II strains and a few type I strains (mainly IB-3 and IC strains) harbor an additional gene in the porphyrin biosynthesis operon, annotated as *deoR* transcriptional repressor (see Fig. S2 and S3 in the supplemental material). The *deoR* gene is located 13 bp upstream of the porphyrin biosynthesis gene cluster. This gene is absent in all acne-associated type IA-2 strains (13). To determine whether *deoR* is functional, we performed gene expression analysis and found that it was expressed in two of the three health-associated type II strains, HL001PA1 and HL042PA3, but not in HL103PA1 (Fig. 6). Characterizations of *deoR*-like repressors in multiple other species have revealed their roles in regulating cellular processes such as sugar phosphotransferase activity, daptomycin production, and fatty acid beta-oxidation (39–41). The close proximity of *deoR* to the porphyrin biosynthesis genes in the genome and the presence and expression of *deoR* in health-associated type II strains suggest that *deoR* may function as a transcriptional repressor in porphyrin biosynthesis. This may partly explain the low porphyrin levels produced by type II strains and their associations with healthy skin.

Acne-associated type IA-2 strains and health-associated type II strains belong to distinct *P. acnes* lineages (11). To further investigate whether the low levels of porphyrins produced by health-associated type II strains were potentially due to repression by *deoR* and not due to other lineage differences, we examined the porphyrin levels produced by a type I *P. acnes* strain, HL025PA1. Strain HL025PA1 represents a distinct lineage within type I and is phylogenetically more similar to type IA-2 strains than to type II strains (see Fig. S3 in the supplemental material). Unlike most other type I strains, HL025PA1 carries and expresses *deoR* in its porphyrin biosynthesis operon (Fig. 6). We found that HL025PA1 produced porphyrins at a level similar to that of type II strains (2.7 µM; *P* = 0.31) (Fig. S4), significantly lower than type IA-2 strains (*P* = 0.0006). Consistent with the trend observed in health-associated type II strains, vitamin B_12_ supplementation resulted in transcriptional repression of the vitamin B_12_ biosynthesis pathway gene in HL025PA1 (Fig. 3), while its porphyrin production was unaffected by vitamin B_12_ supplementation (Fig. S4). This finding further supports an inhibitory role for *deoR* in porphyrin biosynthesis and may potentially explain the health association of type II strains.

10.1128/mSphere.00023-15.2 Figure S2  Porphyrin (*hem*) gene cluster in *P. acnes* strains. All health-associated type II strains harbor a *deoR* transcription repressor (PPA0299, in green) in the porphyrin gene cluster. The number below each box represents the GeneID based on the gene annotation in strain KPA171202. The letters in black boxes indicate the names of the *hem* genes, which are porphyrin biosynthesis genes. PPA0300 and PPA0310 were not assigned a *hem* gene name. Download Figure S2, PDF file, 0.01 MB. Copyright © 2016 Johnson et al. 2016 Johnson et al. This content is distributed under the terms of the Creative Commons Attribution 4.0 International license.

10.1128/mSphere.00023-15.3 Figure S3  Presence of *deoR* in *P. acnes* lineages. *P. acnes* strains carrying *deoR* are shown in green in a phylogenetic tree constructed based on 82 *P. acnes* genomes. All type II strains harbor the *deoR* repressor. Asterisks denote the strains tested in this study. Acne index indicates the strain association with health and disease, as described previously by Fitz-Gibbon et al. (11). Download Figure S3, PDF file, 0.1 MB. Copyright © 2016 Johnson et al. 2016 Johnson et al. This content is distributed under the terms of the Creative Commons Attribution 4.0 International license.

10.1128/mSphere.00023-15.4 Figure S4  Similar to health-associated type II strains, strain HL025PA1 produced a low level of porphyrins and did not respond to vitamin B_12_ supplementation. Strain HL025PA1 was cultured in the medium with (black bars) or without (white bars) vitamin B_12_ (10 µg/ml). Health-associated type II strains, HL103PA1 and HL042PA3, are shown for comparison. Each bar represents the porphyrins produced by each strain normalized to the bacterial culture density. The means plus standard errors (error bars) of data obtained from at least three independent experiments with at least three replicates each are shown. Download Figure S4, PDF file, 0.01 MB. Copyright © 2016 Johnson et al. 2016 Johnson et al. This content is distributed under the terms of the Creative Commons Attribution 4.0 International license.

## DISCUSSION

The role of the human microbiota in disease and health is not yet fully understood. In particular, the molecular mechanisms for different strains of the same commensal species with distinct functions in maintaining health or triggering disease have yet to be elucidated. While the dominant skin commensal, *P. acnes*, is thought to provide protection for skin from colonization of pathogens such as *Staphylococcus aureus* (42, 43), multiple studies have suggested that *P. acnes* can act as an opportunistic pathogen in various diseases, including sarcoidosis, SAPHO syndrome, endodontic lesions, eye infections, prosthetic joint infections, prostate cancer, and acne (1–4). In acne, the dominance of *P. acnes* on both acneic and healthy skin has long been a concern in defining the role of this bacterium in disease pathogenesis (3, 11, 44). Microbiome studies of the skin follicle have revealed strain-level differences of *P. acnes* in health and disease associations (11, 13, 14). However, the molecular mechanisms explaining the strain differences are not well understood.

In *P. acnes*, strain-level differences have been reported at the genomic level (13, 14) and proteomic level (17, 45, 46); however, their molecular link to health and disease remains to be defined. In this study, we investigated the strain-level differences in *P. acnes* at the metabolic level. We discovered that compared to health-associated type II strains, acne-associated type IA-2 strains produced significantly more porphyrins, a group of bacterial metabolites that induce inflammation in acne (Fig. 1). This finding is consistent with previous observations that porphyrin levels were higher in acneic skin than in healthy skin (24–26) and provides one potential molecular mechanism for the role of acne-associated type IA-2 strains in the disease pathogenesis.

This study confirmed our earlier finding that vitamin B_12_ modulates gene expression and metabolic activities of *P. acnes* in acne development (18) and further revealed that acne-associated type IA-2 strains, but not health-associated type II strains, responded to vitamin B_12_ supplementation with increased porphyrin production (Fig. 2). It has been documented that vitamin B_12_ supplementation leads to acne development in a subset of populations (18, 30–33). However, the determinants in individuals who respond to vitamin B_12_ supplementation and develop acne have not yet been identified. Our data show that vitamin B_12_ modulation of porphyrin production is strain specific and suggest that the *P. acnes* strain composition of an individual’s skin microbiota may contribute to vitamin B_12_-induced acne. Individuals harboring acne-associated type IA-2 strains are likely to be at increased risk for developing acne in response to high vitamin B_12_ levels due to the ability of their skin bacteria to produce more porphyrins. On the other hand, individuals whose skin is dominated by health-associated type II strains may have lower porphyrin levels produced by the bacteria, leading to a reduced risk for acne development when supplemented with vitamin B_12_.

This study also revealed a potential transcriptional repression mechanism of porphyrin biosynthesis in health-associated strains through *deoR* regulation (Fig. 6 and see Fig. S2 in the supplemental material). While the function and regulation of *deoR* in *P. acnes* require further investigation, the presence and expression of this gene suggest that *deoR* may play a role in the inhibition of porphyrin production in health-associated type II strains. *P. acnes* strain HL025PA1, although belonging to type I, represents a distinct lineage. It carries and expresses *deoR*. Lomholt and Kilian previously reported that only 1 of their 13 studied *P. acnes* isolates from this lineage (sequence type 27 [ST27], as designated by the Aarhus MLST_9_ scheme) was from severe acne and suggested an association of this lineage with healthy skin (12). This observation supports our theory of a role for *deoR* in skin health. As *deoR* expression was not detected in all tested health-associated type II strains, additional mechanisms regulating porphyrin production likely exist, warranting future investigation. The low levels of porphyrins produced in health-associated strains, especially strain HL001PA1, make these strains candidates for topical probiotics to potentially modify an acne-prone skin microbial community and return the skin to a healthy state. This could be a new strategy in future acne therapeutics.

Our current analysis is focused on two major *P. acnes* lineages, types IA-2 and II, which have been associated with acne and healthy skin, respectively. Further investigations of metabolic activities and their regulations including porphyrin production in strains from other lineages will shed light on additional bacterial factors contributing to disease or health.

Our study has implications in the development of novel acne therapies. Current acne treatments, such as antibiotics and retinoids, are often ineffective and can have adverse side effects. Moreover, the use of antibiotics has led to the emergence of antibiotic-resistant strains and an increase in treatment failure (47, 48). Although new acne therapeutics have been in demand for a long time, the unclear etiology of the disease has crippled the design of new and effective treatments over the past 3 decades. We demonstrated that LA at the concentration of 0.1 mg/ml effectively inhibited porphyrin biosynthesis in *P. acnes* strains (Fig. 5). This inhibition is consistent with the decreased porphobilinogen synthase activity observed in *Pseudomonas aeruginosa* upon LA treatment *in vitro* (38). LA is also known to inhibit bacterial growth; thus, a suboptimal concentration was used in this study to demonstrate the ability of this molecule to effectively reduce the porphyrin levels produced by *P. acnes* without significant inhibition of bacterial growth. In our study, we aimed to model a treatment strategy in which the virulence of acne-associated strains is targeted without disrupting the growth of health-associated strains. Other compounds, such as 4,6-dioxoheptanoic acid and isonicotinic acid hydrazide, also inhibit porphobilinogen synthase (49, 50) and can potentially be used to inhibit porphyrin biosynthesis. LA and other related small molecules that inhibit porphyrin biosynthesis in *P. acnes* are attractive drug candidates for the treatment of acne.

The benefit of the microbiota to human health is increasingly recognized. There is a need to improve our current approaches in treating microorganism-associated diseases. Most of the available approaches nonspecifically target the microbiota using broad-spectrum antibiotics and antimicrobials, potentially leading to a disruption in the colonization of beneficial microorganisms. This study presents an advance toward a better understanding of the beneficial microbiota and targeted therapeutics. By investigating the molecular mechanisms underlying the differences between health- and disease-associated strains, we suggest that inhibiting disease-associated strains with specific targets while maintaining or supplementing health-associated strains can potentially be a new strategy in the future for treating microorganism-associated diseases.

## MATERIALS AND METHODS

### *P. acnes* strains and cultures.

*P. acnes* strains used in this study (Table 1) were isolated previously as described by Fitz-Gibbon et al. (11). Briefly, each strain was isolated from the contents collected from multiple hair follicles on the nose of an acne patient or healthy individual. The sampled skin site of acne patients may or may not have visible acne lesions; therefore, the strains do not necessarily correspond to the diseased or healthy state. Four RT4 and RT5 *P. acnes* strains, HL053PA1, HL045PA1, HL043PA1, and HL043PA2, were selected to represent type IA-2 strains, which were associated with the disease based on a 16S rRNA metagenomic study (11). Like most of the RT4 and RT5 strains, these strains harbor mutations in their 16S and 23S rRNA genes, which confer antibiotic resistance. Three RT2 and RT6 strains, HL001PA1, HL103PA1, and HL042PA3, were selected to represent type II strains that were associated with healthy skin (10–12, 15). To confirm the findings, three other type II strains, HL110PA3, HL106PA1, and HL050PA2, were also tested. The genome sequences of these 10 strains were reported previously (11, 13). For each experiment, 5 ml of reinforced clostridial broth was inoculated with 5 × 10^5^ *P. acnes* cells per ml of culture. Cultures were grown to stationary phase anaerobically at 37°C in a light-protected box. Cultures were supplemented on day 0 with vitamin B_12_ (10 µg/ml), and/or 5-ALA (0.1 mg/ml), and/or LA (0.1 mg/ml). For controls, *P. acnes* strains were also cultured without supplementation. Three to five independent experiments with at least three replicates per experiment were performed for each strain, except for strains HL110PA3, HL106PA1, and HL050PA2, which were tested in only one experiment with three replicates.

### Extraction, identification, and quantification of extracellular porphyrins.

For each strain, porphyrins were extracted by the method of Kang et al. (18). Briefly, 500 µl of bacterial culture was extracted in ethyl acetate and acetic acid (4:1, vol/vol) and solubilized in 1.5 M HCl. The absorbance at 405 nm was measured from 200 µl of the soluble phase using a Tecan Genios spectrophotometer M1000 (Tecan U.S. Inc., Morrisville, NC). The standard curve to convert absorbance to concentration was generated using coproporphyrin III standards of known concentration (catalog no. C654-3; Frontier SCI). We measured the porphyrin level in *P. acnes* strain KPA171202 using our porphyrin extraction method and the quantification method of Wollenberg et al. (36). Our measurement, which was 206 pmol/mg, is consistent with the reported value of 220 pmol/mg by Wollenberg et al. (36). This indicates that our method is comparable to the methods in previous studies. Porphyrins were extracted from cultures at stationary phase. The results from cultures at mid-log phase had a consistent trend found in cultures at stationary phase. Bacterial culture density was measured at an optical density at 595 nm (OD_595_) for normalization of porphyrin levels. For mass spectrometry experiments, extracted porphyrins were directly injected onto an Agilent 6460 triple quadrupole liquid chromatography-mass spectrometry (LC/MS) system, and each strain’s *m*/*z* in the negative-ion mode was identified.

### Statistical analysis for porphyrin production comparisons.

The average amount of porphyrins produced by each strain under each culture condition was calculated based on the data from at least three independent experiments, with at least three replicates for each. The porphyrin levels between strains, groups (acne-associated versus health-associated), and culture conditions (with treatment versus without treatment) were estimated in a linear mixed-effect model, with random intercepts by trial to account for trial effects. *P* values for specific comparisons among groups were corrected using Tukey’s method. All statistical analysis was performed using R software (version 3.1.3).

### Identification of *deoR* transcription repressor.

The sequences of the porphyrin gene clusters from 82 *P. acnes* genomes (13) were aligned using the multiple sequence alignment software (Clustal W2) (51). The *deoR* transcription repressor (PPA0299) was identified as an extra genomic element found in all health-associated type II strains and a few type I strains.

### RNA extraction and cDNA synthesis.

For *cbiL* and *deoR* gene expression analysis, cells were lysed with bead beating. Total RNA was extracted using the standard phenol-chloroform method and purified using RNeasy kit (Qiagen). DNA was removed using the Turbo DNA-free kit (Life Technologies). RNA quality was assessed using gel electrophoresis. Single-stranded cDNA was synthesized using SuperScript III first-strand synthesis supermix (Life Technologies).

### Analysis of *cbiL* gene expression.

qRT-PCR was performed using the LightCycler 480 high-resolution melting master mix (Roche) on a LightCycler 480 instrument (Roche) with the following primers: *cbiL*-forward, 5′ GCGCGAGGCAGACGTGATCC 3′, and *cbiL*-reverse, 5′ GACACCGGACCTCTCCCGCA 3′. The following qRT-PCR protocol was used: initial denaturation at 95°C for 5 min, followed by 50 cycles, with 1 cycle consisting of 95°C for 10 s, 62°C for 30 s, and 72°C for 30 s. The fold change in *cbiL* gene expression between cultures with vitamin B_12_ supplementation and cultures without supplementation was calculated. The gene expression level of *cbiL* in each sample was normalized against the 16S rRNA transcript level. Melting curve analysis was performed using the LightCycler 480 software version 1.5 (Roche) to verify the specificity of the amplified products based on their melting temperatures. All reactions were run in triplicate.

### Analysis of *deoR* gene expression.

*deoR* was amplified from the cDNA and genomic DNA of several *P. acnes* strains using a C1000 thermal cycler (BioRad). The following primers were used in the PCR: *deoR*-forward, 5′ CTGGCACGAGAAGGAACAA 3′, and *deoR*-reverse, 5′ GAATCGAGCAGAACTAGGTCAC 3′. The following PCR protocol was used: initial denaturation at 95°C for 5 min, followed by 35 cycles, with 1 cycle consisting of 95°C for 10 s, 62°C for 30 s, and 72°C for 30 s, followed by one cycle at 72°C for 5 min. Amplified *deoR* products were visualized on a 2% agarose gel. The amplification of 16S rRNA was included as a positive control. Acne-associated strain HL045PA1 was used as a negative control for *deoR* expression.
