# Supplementary figures and images for: Strain-Level Differences in Porphyrin Production and Regulation in Propionibacterium acnes Elucidate Disease Associations
Source: mSphere. 2016 Feb 10;1(1):e00023-15. doi: 10.1128/mSphere.00023-15 (PMC4863617; doi:10.1128/mSphere.00023-15)

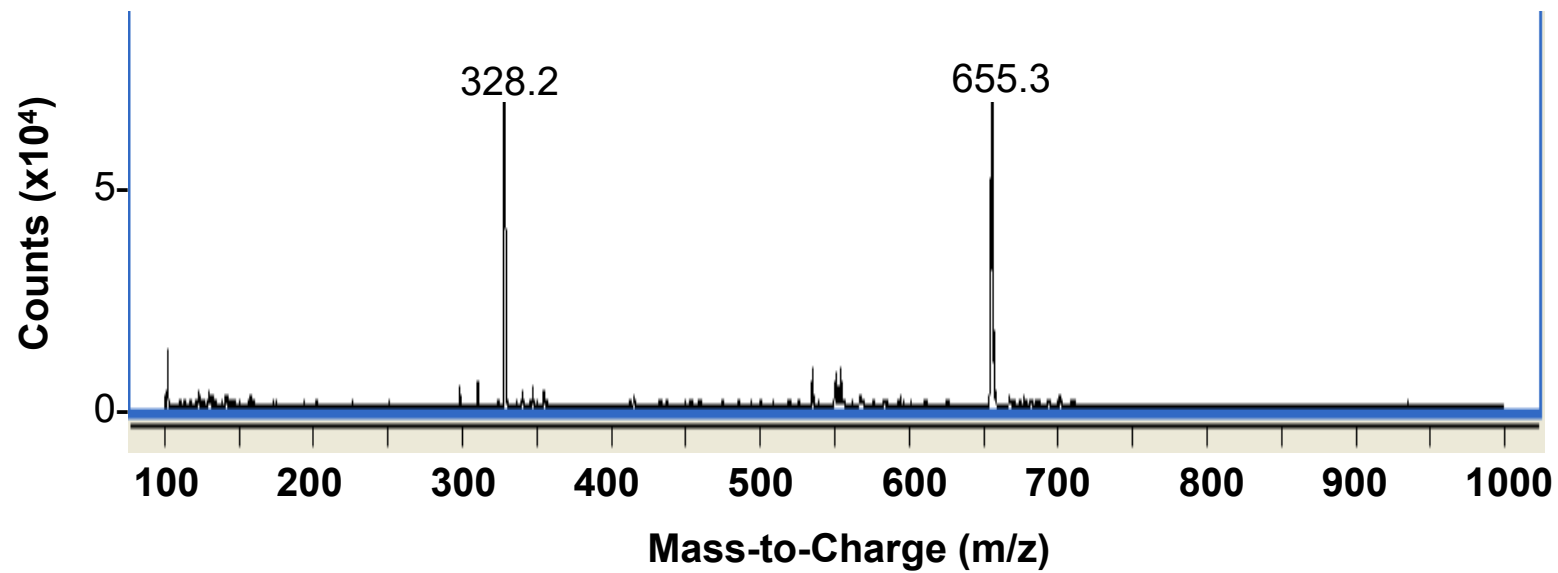

Supplement: Figure S1 [file sph001162006sf1.pdf]

### Health-associated type II strains

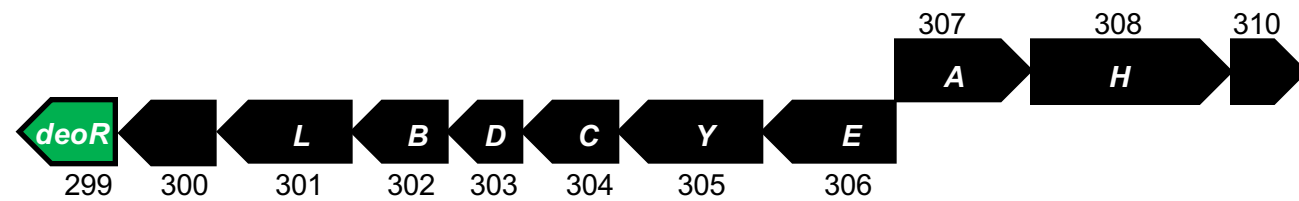

### Acne-associated type IA-2 strains

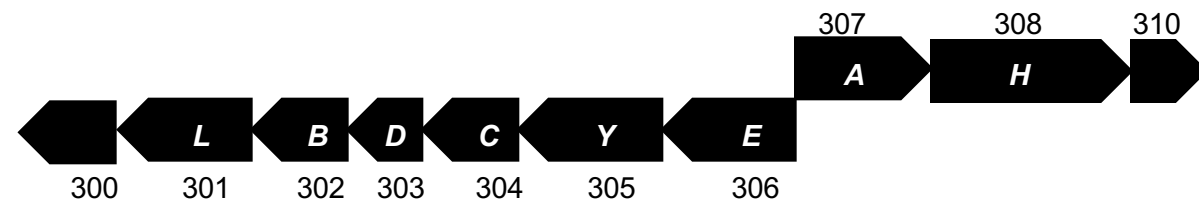

Supplement: Figure S2 [file sph001162006sf2.pdf]

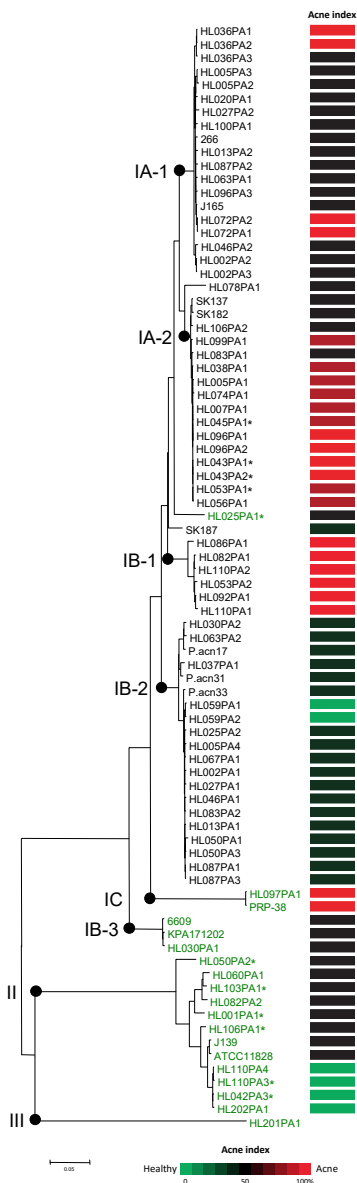

Supplement: Figure S3 [file sph001162006sf3.pdf]

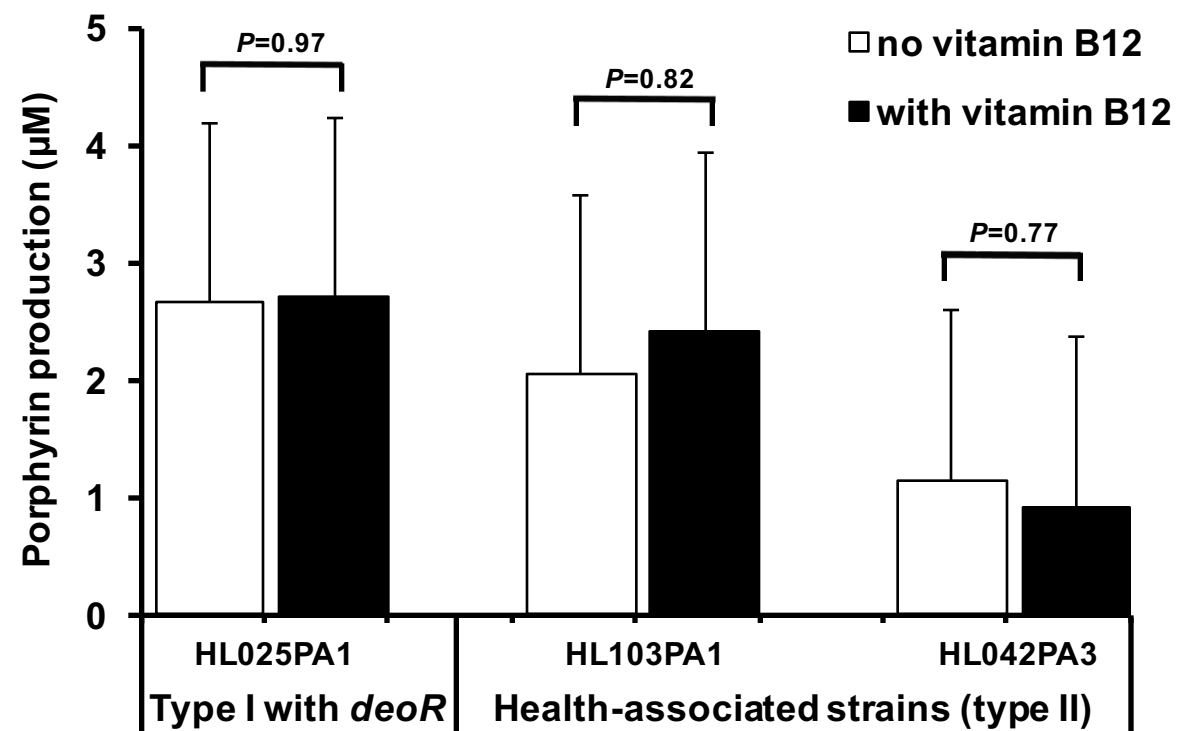

Supplement: Figure S4 [file sph001162006sf4.pdf]
